# A New Generation of Eco-Designed Embolic Agents: Towards Sustainable and Personalized Interventional Radiology

**DOI:** 10.3390/jpm16020064

**Published:** 2026-01-29

**Authors:** Alexis Ruimy, Thibault Agripnidis, Julien Panneau, Johanna Nguyen, Farouk Tradi, Thierry Marx, Raphaël Haumont, Pauline Brige, Benjamin Guillet, Vincent Vidal

**Affiliations:** 1 Interventional Radiology Section, Department of Medical Imaging, University Hospital Timone, AP-HM, 13005 Marseille, France; thibault.agripnidis@ap-hm.fr (T.A.); julien.panneau@ap-hm.fr (J.P.); farouk.tradi@ap-hm.fr (F.T.); vincent.vidal@ap-hm.fr (V.V.); 2 Aix Marseille University, LIIE, 13005 Marseille, France; johanna.nguyen@univ-amu.fr (J.N.); pauline.brige@univ-amu.fr (P.B.); 3 Aix Marseille University, CERIMED, 13005 Marseille, France; benjamin.guillet@ap-hm.fr; 4 Aix-Marseille University, INSERM 1263, INRAE 1260, C2VN, 13005 Marseille, France; 5 TM4, 91000 Paris, France; t4marx@aol.com; 6 Laboratoire de Physico-Chimie de l’Etat Solide, ICMMO, CNRS-UMR 8182, Bâtiment 410-Université Paris-Sud XI, 15 rue Georges Clémenceau, 91405 Orsay, France; raphael.haumont@u-psud.fr; 7 Department of Nuclear Medicine, Assistance Publique-Hôpitaux de Marseille (AP-HM), 13385 Marseille, France

**Keywords:** arterial embolization, embolic agent, hemorrhage, organic agent, agar

## Abstract

**Background:** Embolization is a key therapeutic option in interventional radiology for the management of acute arterial bleeding and solid organ injuries. While various embolic agents exist, there is a persistent clinical need for materials that are not only highly effective but also biocompatible, easy to deliver, and cost-effective. We aim to evaluate a new eco-friendly dry foam agar-based embolization agent (ABEA) in an uncontrolled solid organ hemorrhage model. **Material and Methods:** Ten pigs underwent a controlled splenic injury. After a 5 min free-bleeding period, five pigs were treated with splenic artery ABEA embolization, while the remaining five received no treatment and served as the control group. Follow-up angiography was performed immediately after embolization and at 5 and 15 min in the treated pigs. Mean arterial pressures and average blood loss volumes were evaluated for 120 min. **Results:** The control group showed continuous blood loss, leading to a significantly higher total blood loss than the ABEA-treated group (1451 mL vs. 611 mL at 120 min, *p* < 0.05). Mean arterial pressure (MAP) remained below the hemorrhagic shock threshold throughout the procedure in the control group, validating the model of uncontrolled hemorrhage. In addition, a significant stabilization of MAP was observed in treated pigs, remaining above the critical level of hemorrhagic shock and differed significantly from control group values. **Conclusions:** Embolization with ABEA maintained MAP above critical levels and significantly reduced blood loss volume in a hemorrhagic model. These results support the technical feasibility and short-term hemostatic performance of ABEA in an acute setting. While preliminary, this proof-of-concept has provided the basis for a validated clinical study currently underway to evaluate its effectiveness and safety in human patients.

## 1. Introduction

Embolization represents a cornerstone technique in interventional radiology (IR), used in both elective and emergency settings where rapid hemostasis for uncontrolled hemorrhage can be lifesaving [1]. The management of major arterial bleeding and solid organ injuries through these minimally invasive techniques is essential to prevent complex and potentially morbid open surgical procedures [2,3]. However, conventional embolic agents such as metallic coils, gelatin sponge, and liquid embolics including n-butyl cyanoacrylate (NBCA) or polymers present notable limitations [4]. Coils are expensive and may cause delayed occlusion; gelatin sponge is animal-based and undergoes unpredictable resorption; liquid agents are technically demanding, sometimes toxic, and produced from non-renewable sources [5,6]. These constraints underline the need for next-generation materials that combine efficacy, safety, and affordability. In line with the recent evolution of IR toward more personalized and sustainable medicine [7], there is also a growing societal expectation for materials that are ecologically responsible.

To address these challenges, our research has focused on developing an innovative agar-based embolization agent (ABEA). This agent, derived from plant-based agar, is designed to be an efficient and easy-to-use alternative that is also environmentally sustainable. The primary objective of this proof-of-concept preclinical study is to evaluate the hemostatic efficacy of a new dry-foam ABEA in a swine model of uncontrolled solid organ hemorrhage. While its feasibility has already been proven in previous arterial embolization models [8], its performance in critical, real-world bleeding scenarios remains to be established.

Existing animal models of uncontrolled hemorrhage often rely on extreme scenarios combining massive blood loss, coagulopathy, and aggressive resuscitation protocols—conditions that do not fully reflect the clinical presentation of most patients undergoing emergency embolization [9,10]. In real-world practice, bleeding is often partially controlled, and hemodynamic instability is present but not uniformly associated with profound coagulopathy.

By using a refined swine model designed to more closely approximate this clinical reality, we seek to assess the impact of ABEA on blood loss and hemodynamic stability. Through this work, we aim to explore the potential of this innovative, eco-designed embolic agent in the future landscape of modern, personalized, and environmentally responsible interventional radiology.

## 2. Materials and Methods

### 2.1. Animal Preparation

Ten Pietrain pigs (females and males, 35 kg ± 5) were used following approval by the Ethical Animal Committee (APAFIS referral number #455596), in accordance with EU Directive 2010/63/EU for animal experiments. This study was designed primarily as a feasibility and proof-of-concept experiment. Given its exploratory nature and the limited number of animals, no formal power calculation was performed; therefore, the results should be interpreted as preliminary data. All invasive procedures were performed under general anesthesia and according to strict aseptic conditions. After intramuscular sedation with 20 mg kg^−1^ ketamine and 0.11 mg kg^−1^ acepromazine, the animals were placed in dorsal recumbent position. Intravenous access and blood samples were obtained through a venous catheter inserted into an auricular vein. Induction of anesthesia was obtained by 2 mg kg^−1^ propofol through the venous catheter and maintained with 2% sevoflurane gas by mechanical ventilation (Dräger Zeus^®^, Dräger Inc., Telford, PA, USA). Analgesia was obtained by continuous 24 µg kg^−1^·h^−1^ fentanyl perfusion.

Aseptic percutaneous arterial access was obtained under ultrasound guidance, with placement of a left common carotid artery catheter for continuous invasive blood pressure monitoring. For 2 h, we conducted an assessment of the following hemodynamic parameters at regular 15 min intervals: arterial pressure, end-tidal carbon dioxide (EtCO_2_), heart rate real-time monitoring using electrocardiogram electrodes and peripheral oxygen saturation (SpO_2_) using a pulse oximeter.

### 2.2. Laparotomy and Splenic Preparation

After spleen exposure by laparotomy, a double ligature was made on the left gastroepiploic branch at two sites approximately 2 cm apart. The organ was exteriorized and placed longitudinally on the open abdomen, lightly placed on a slope to facilitate blood collection within a plastic sheet surrounded by pre-weighed gauze pads to ensure accurate collection of shed blood from the forthcoming splenic injury. It should be noted that this extracorporeal splenic hemorrhage model differs substantially from real-life clinical scenarios where the organ remains in situ. However, this experimental constraint was necessary to ensure the most precise monitoring and quantification of blood loss throughout the procedure.

### 2.3. Endovascular Preparation

Arterial access was established via the common femoral artery using the standard Seldinger technique under ultrasound guidance. Following the placement of a 6F vascular sheath, a 5F Cobra Wirebraid catheter (Cordis, Fremont, CA, USA) was navigated through the iliac arteries and the abdominal aorta. The selection of the celiac trunk and the subsequent catheterization of the splenic artery were performed under continuous fluoroscopic guidance (radioscopy) to ensure real-time visualization of the catheter trajectory and to avoid any intimal injury. To confirm the precise selective positioning of the catheter tip and to map the individual vascular anatomy of each subject, manual injections of iodinated contrast medium were performed intermittently. Once the proximal splenic artery was successfully selected, a baseline digital subtraction angiography (DSA) was obtained using a mobile C-arm system (OEC One CFD, General Electric Medical System, Minneapolis, MN, USA) to document the pre-injury parenchymal enhancement and vascular flow. The catheter was then secured and maintained in this stable position throughout the entire experimental period in both the control and treated groups, allowing for immediate intervention and consistent follow-up imaging.

### 2.4. Splenic Injury and Embolization

**Figure 1:**
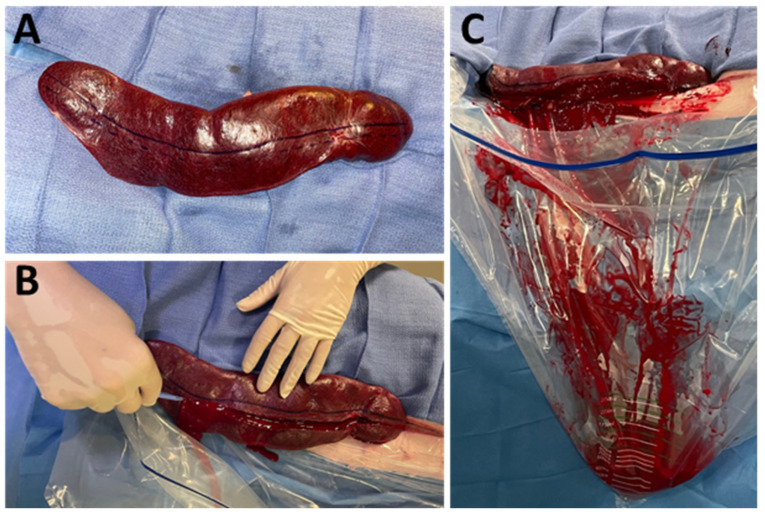
Exteriorization and section of the spleen. (**A**): Exteriorized spleen; the solid blue line represents splenic midline, the dashed line represents planned transection; (**B**): splenic transection along the dashed line; (**C**): active shed blood collection after splenic transection recovered in the hemorrhage collection bag.

**Figure 2:**
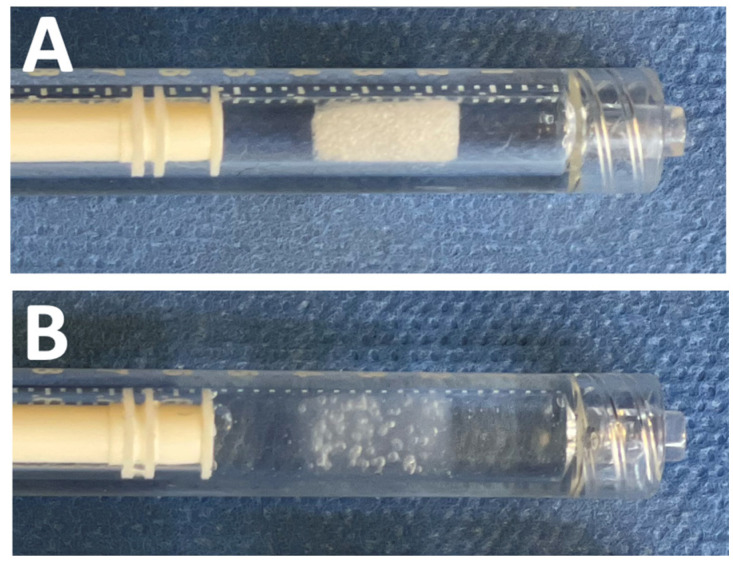
ABEA (Agar-Based Embolization Agent) implant in a 1 mL syringe: Dry foam implant (**A**) and hydrated implant in saline solution (**B**).

After marking the longitudinal midline of the spleen, the organ was sharply and completely transected 1 cm lateral to the midline, as described by Sondeen et al. (Figure 1) [11]. Free splenic hemorrhage was allowed for 5 min, then the catheter was advanced into the proximal splenic artery over the wire for angiography and treatment. The blood was collected continuously and measured every 15 min in collection bags. Animals were randomly assigned to control or treatment groups. Five pigs were in the control group with no treatment performed, while five pigs underwent treatment by an endovascular embolization with ABEA implants. For each embolization, an ABEA implant (5 × 4 × 10 mm) was directly inserted into an opened 1 mL syringe, followed by aspiration of 1 mL of saline solution. (Figure 2) The syringe was then connected to the catheter and flushed allowing the implant to be positioned within the catheter dead space. Iodinated contrast medium was subsequently injected, and the implant expulsion was monitored under fluoroscopy. The injection endpoint was defined as complete angiographic occlusion of the splenic artery by using as many implants as necessary. Follow-up angiography was systematically performed at 5 and 15 min after ABEA embolization to assess vessel occlusion and embolic material position. After 2 h of follow-up, the pigs were euthanized with 180 mg kg^−1^ pentobarbital injected through the venous auricular catheter. The spleen was explanted for macroscopic analysis of the embolized splenic artery.

### 2.5. Data Analysis

The study was designed to assess the treatment response compared to the control group. Mean arterial pressures, average blood loss volumes, and other relevant measures were determined using analysis of variance with multiple comparisons, followed by appropriate post hoc tests using GraphPadPrism Software 8 (GraphPad, Boston, MA, USA). Data are presented as mean ± standard error of the mean. Significance was established for a *p*-value less than 0.05. The primary endpoints, determined a priori, were to demonstrate a significant difference in terms of blood loss volume and mean arterial pressure (MAP) between the treated and control groups. As no formal power calculation was performed prior to the study, these statistical results are intended to provide preliminary proof-of-concept evidence rather than definitive confirmation of effect size.

## 3. Results

### 3.1. Baseline Homogeneity and Hemorrhagic Model Validation

Prior to the induction of splenic injury, there were no significant differences between the control and ABEA groups regarding baseline physiological parameters. The mean weights and initial heart rates were comparable across all ten subjects, ensuring a homogeneous starting point for the study. All animals in the control group (*n* = 5) reached a mean arterial pressure (MAP) below 60 mmHg, with an average over time measured at 50 mmHg (±5.1). In this group, continuous blood loss was recorded throughout the 120 min procedure, reaching an average of 1450 mL, which represents nearly 50% of their total blood volume.

### 3.2. Embolization Procedure

**Figure 3:**
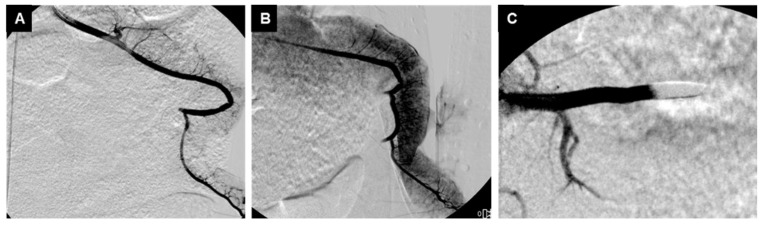
Angiographic controls before (**A**), after (**B**) splenic section, and after embolization with ABEA (Agar-Based Embolization Agent) (**C**).

Technical success, characterized by the precise delivery and stable positioning of the ABEA implants, was achieved in 100% of the intervention group (*n* = 5). Follow-up digital subtraction angiography (DSA) performed immediately post-embolization confirmed complete and immediate proximal occlusion of the splenic artery in all treated pigs. In vessels ranging from 3 to 4 mm, a single implant was sufficient to stop the flow. However, the procedural versatility was highlighted in a subject with a 5.5 mm artery, where a sequential deployment of six implants successfully achieved stasis by effectively blocking the downstream bed and providing a scaffold for the proximal occlusion. Angiographic controls at 5 and 15 min post-intervention showed no evidence of distal migration or fragmented material, suggesting high-mechanical stability of the hydrated agar foam under arterial pressure in an acute hemorrhagic setting (Figure 3).

Individual heart rate, blood loss, and arterial pressure values for each animal are reported in the comprehensive results table and are provided as Supplementary Table S1.

### 3.3. Blood Loss

**Figure 4:**
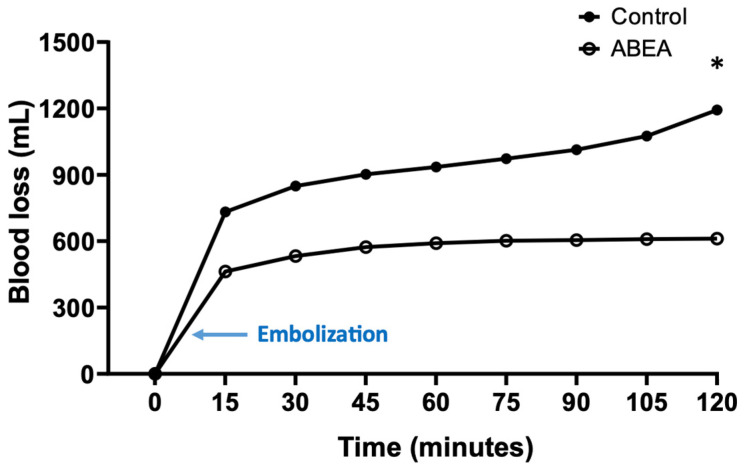
Cumulative blood loss over time in control and embolized with ABEA pigs. * Significant difference between the two groups (*p* = 0.03).

In all animals, the splenic injury caused immediate and abundant hemorrhage. Following treatment, a reduction in blood loss was observed, whereas hemorrhage appeared to persist in the control group (Figure 4). The divergence in blood loss between the two study groups became statistically apparent at the initial 15 min measurement and continued to widen significantly throughout the 120 min observation period. Animals in the control group demonstrated a continuous and near-linear increase in cumulative blood loss. By the end of the 120 min procedure, the mean total loss reached 1451 mL (±486 mL), which represents nearly 50% of the total estimated blood volume for pigs in this weight range. This significant increase from the 15 min mark (732 mL ± 187 mL) to the final measurement was statistically confirmed with a *p*-value of 0.03. In contrast, the ABEA-treated group exhibited a rapid stabilization of blood loss almost immediately following the embolization procedure. The cumulative blood loss curve effectively reached a plateau, with no significant statistical difference recorded between the 15 min mark (462 mL ± 146 mL) and the final 120 min measurement (611 mL ± 161 mL, *p* = 0.99). At the conclusion of the 120 min observation, the average cumulative blood loss of 1451 mL in untreated pigs was significantly higher than the 611 mL recorded in embolized pigs (*p* = 0.03), within the limitations of this pilot cohort.

### 3.4. Blood Pressure

**Figure 5:**
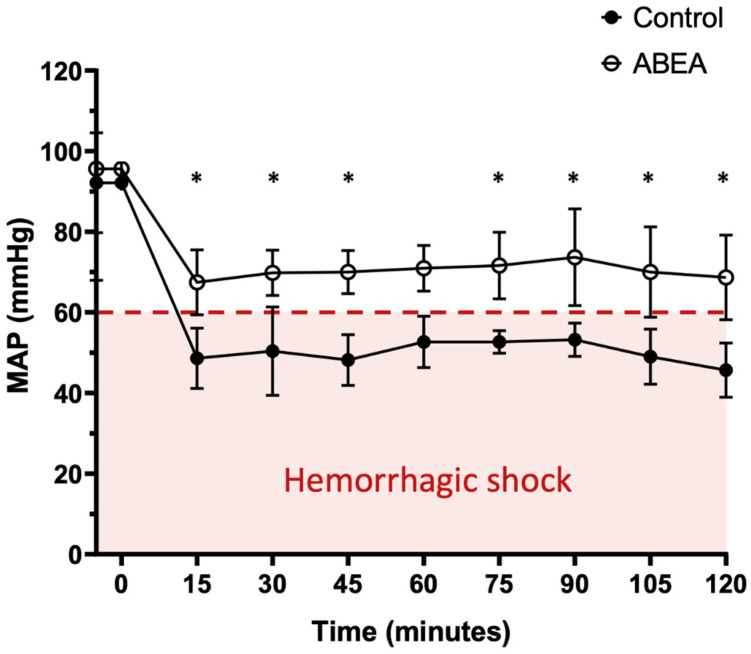
Mean arterial pressure (MAP) over time after establishment of the hemorrhagic model in control and ABEA-embolized groups. * significant difference between the groups (*p* < 0.05).

Blood pressure decreased in all groups during the first 15 min. While all animals in the control group reached values below the 60 mmHg MAP threshold, the embolized group maintained values above this threshold throughout the 120 min observation period. A significant difference was observed at almost all-time points between the two groups (*p* < 0.05), except for the 60 min measurement. Specifically, the treated pigs maintained a mean arterial pressure (MAP) of 70.3 mmHg ± 6.4, compared to untreated pigs, whose mean MAP was 50 mmHg ± 5.1 (Figure 5).

### 3.5. Cardiac Frequency

Heart rate measurements were recorded at regular intervals to evaluate the compensatory reaction to blood loss. There was a progressive and persistent increase in the heart rate of pigs in the control group over the 120 min study period. The pigs treated with ABEA showed a less pronounced increase in heart rate compared to the untreated animals. At the conclusion of the 120 min observation period, the mean heart rate was 170.8 bpm for the control group and 139.4 bpm for the ABEA-treated group. Despite this numerical difference, no overall significant statistical difference in heart rate was established between the two groups during the procedure. A statistically significant difference in heart rate between the control and treatment groups was only transiently observed at the 45 min mark (*p* < 0.05).

### 3.6. Post-Mortem Macroscopic Findings

**Figure 6:**
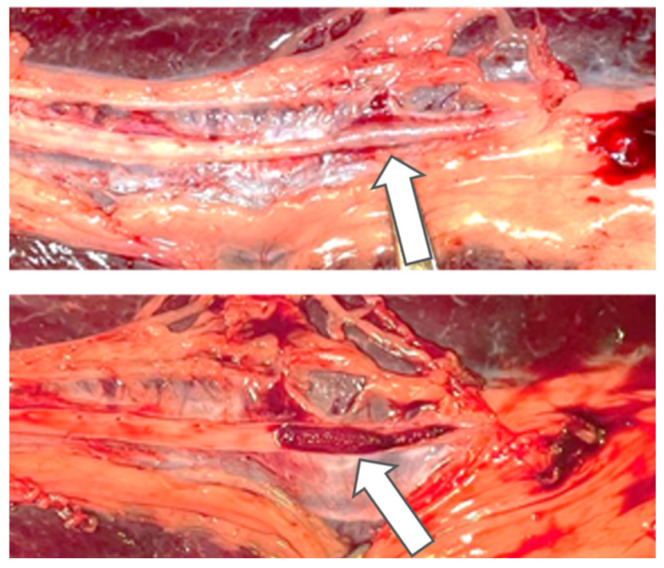
Photographs of macroscopic analyses of the splenic artery embolized with ABEA (Agar-Based Embolization Agent, arrow).

Post-mortem macroscopic analysis of the explanted spleens provided further qualitative evidence regarding the performance and positioning of the embolic material within the vascular system. Upon dissection and longitudinal opening of the splenic artery, the ABEA implants were found to remain securely in place. Visual inspection of the arterial wall in contact with the implants revealed that the material conformed to the lumen after dissection. The post-mortem findings supported the angiographic evidence of complete vessel occlusion achieved during the live phase of the experiment, with the implants successfully maintaining their position without evidence of distal migration (Figure 6).

## 4. Discussion

Conventional embolic agents, such as metallic coils or liquid polymers, are often derived from non-renewable resources, and their manufacturing processes are energy-intensive. Furthermore, the long-term presence of non-degradable plastics or metals in the body presents ongoing environmental and biological considerations. One of the most innovative aspects of ABEA is its alignment with the principles of sustainable interventional radiology [12]. ABEA, as an organic compound derived from plant-based agar, offers a sustainable alternative. Agar is a naturally occurring polysaccharide with a well-established safety profile. Its production requires minimal energy input compared to synthetic polymers. By eliminating the reliance on plastics and difficult-to-extract metals, ABEA addresses a growing societal expectation for implantable medical devices with a low environmental impact. This allows for life-saving medical interventions to be performed while minimizing the ecological footprint of the procedure. Biological safety is a paramount concern for any implantable medical device. Agar–agar has been utilized for decades in food and pharmaceutical industries, which underscores its inherent biological neutrality [13].

ABEA had already been studied in an article as a chronic embolization agent [8]. We wanted to evaluate its utilization in a hemorrhagic model. Embolizations were performed simply and quickly with a product that is ready to use, with fast preparation time and fast effective vessel occlusion. A proximal occlusion was achieved using ABEA implants, resulting in a complete cessation of splenic artery blood flow in all pigs. While most pigs (5/6) required only one or two ABEA implants to achieve immediate occlusion of the splenic artery, one pig required six. The splenic artery trunk of this particular pig measured 5.5 mm in diameter. Although the 5 mm implant is ideally designed for vessels smaller than 3 mm, this case demonstrated that it can also be used in larger arteries if the downstream vascular network is preliminarily blocked with implants.

The physical modularity of ABEA was further investigated in a recently published study [14]. This research explored the feasibility of manually bisecting the ABEA implant longitudinally to accommodate smaller vascular calibers. The study demonstrated that these halved segments remained structurally intact during their passage through a microcatheter and upon delivery into the target vessel. Despite the reduction in size, the agent maintained its physical integrity and ensured effective, permanent occlusion. This finding is crucial for personalized interventional medicine, as it proves that the clinician can manually tailor the implant to fit specific anatomical requirements in rea; time, expanding the therapeutic range of the device to include smaller arterial branches without compromising its hemostatic performance.

With ABEA, blood loss is limited and stabilizes from the 15th minute after the onset of hemorrhage. While the use of ABEA allowed for the maintenance of mean arterial pressure (MAP) above 60 mmHg, the control group’s pressure remained consistently below this threshold. This is a critical observation, as a MAP lower than 60 mmHg is recognized as the threshold for hemorrhagic shock [15]. Ensuring a significant difference from the control group at almost every measurement suggests that ABEA provides effective early hemodynamic stabilization in this acute model.

Our approach differed significantly from the Sondeen et al. reference study [11]. In their study, an initial 30% total blood volume depletion was initiated before embolization, and they used fluid resuscitation immediately after embolization. Moreover, they induced a coagulopathy with hypothermia and anticoagulation in 50% of the animals. In their study, 100% of the control groups died within two hours, while our results revealed that all our subjects survived the full two hours. This difference raises the question of the relevance of the mortality criterion in this context. Indeed, our model seems to better represent the clinical reality, where severely hemorrhaging patients often arrive at the hospital with semi-controlled arterial injuries. However, it must be noted that the hemodynamic stabilization observed here represents a short-term physiological response and should not be directly extrapolated to long-term clinical survival, although the embolic durability of ABEA—with persistent occlusion and biocompatibility at three months—has already been confirmed in a previous chronic model [8].

Despite these encouraging preliminary results, several significant limitations must be addressed to put our findings into perspective. First, the small sample size (*n* = 5 per group) reflects a deliberate ethical choice to minimize animal usage in accordance with the “Reduction” principle of the 3Rs, as this was a feasibility and proof-of-concept study. Consequently, the lack of a formal power calculation means that these results should be interpreted as preliminary data. Second, no direct comparative efficacy analysis with existing embolic agents was performed, making any comparison with current standards speculative. Third, the observation period was limited to 120 min to focus specifically on the acute hemostatic phase. While this does not allow for the assessment of delayed re-bleeding in this specific model, the chronic efficacy of ABEA—showing persistent occlusion up to three months—has already been demonstrated in previous published work [8]. Fourth, defining shock solely by MAP omits metabolic markers (e.g., lactate, base deficit), limiting the assessment of physiologic recovery versus only hemodynamic stabilization. Finally, the experimental conditions do not fully replicate clinical scenarios, particularly in the extracorporeal positioning of the spleen. This positioning does not allow for intra-abdominal tamponade, although it enabled the collection of all shed blood without interruption for a stringent assessment of the hemostatic effects.

Agar is a naturally derived polysaccharide that has been widely used for decades in the food industry and as a dietary supplement. Unlike most commercially available agents, ABEA can be designated as an organic compound due to its sustainable sourcing and environmentally friendly production process, which requires minimal energy input. Moreover, by eliminating the use of plastics and difficult-to-extract metals, ABEA aligns with the future landscape of modern, personalized, and environmentally responsible interventional radiology.

## 5. Conclusions

We have demonstrated the technical feasibility and preliminary efficacy of this new agar-based embolization agent (ABEA) in a pilot translational hemorrhagic model, noting its ability to maintain hemodynamic parameters and achieve rapid vessel occlusion. While these initial results should be interpreted as a proof-of-concept, a clinical study has already been validated and is currently underway to further evaluate its performance and safety in a clinical setting. Beyond its initial therapeutic performance, ABEA embodies the principles of sustainability, accessibility, and ethical responsibility, offering a promising perspective for the next generation of eco-designed implantable medical materials in interventional radiology.

## Data Availability

The original contributions presented in this study are included in the article material. Further inquiries can be directed to the corresponding author.

## Supplementary Materials

The following supporting information can be downloaded at: https://www.mdpi.com/article/10.3390/jpm16020064/s1, Table S1: Individual heart rate, blood loss, and arterial pressure values for each animal.

## Abbreviations

The following abbreviations are used in this manuscript:

ABEA	Agar Based Embolization Agent
IR	Interventional Radiology
